# Cortical hemodynamics as a function of handgrip strength and cognitive performance: a cross-sectional fNIRS study in younger adults

**DOI:** 10.1186/s12868-021-00615-6

**Published:** 2021-02-15

**Authors:** Fabian Herold, Tom Behrendt, Alexander Törpel, Dennis Hamacher, Notger G. Müller, Lutz Schega

**Affiliations:** 1 Department of Neurology, Medical Faculty, Otto Von Guericke University, Leipziger Str. 44, 39120 Magdeburg, Germany; 2 Research Group Neuroprotection, German Center for Neurodegenerative Diseases (DZNE), Leipziger Str. 44, 39120 Magdeburg, Germany; 3 Institute III, Department of Sport Science, Otto Von Guericke University Magdeburg, Zschokkestr. 32, 39104 Magdeburg, Germany; 4 Center for Behavioral Brain Sciences (CBBS), Brenneckestraße 6, 39118 Magdeburg, Germany

**Keywords:** Cognition, Functional near-infrared spectroscopy, Handgrip strength, Cognition, Sternberg paradigm, Muscular fitness

## Abstract

**Background:**

There is growing evidence for a positive correlation between measures of muscular strength and cognitive abilities. However, the neurophysiological correlates of this relationship are not well understood so far. The aim of this study was to investigate cortical hemodynamics [i.e., changes in concentrations of oxygenated (oxyHb) and deoxygenated hemoglobin (deoxyHb)] as a possible link between measures of muscular strength and cognitive performance.

**Methods:**

In a cohort of younger adults (n = 39, 18–30 years), we assessed (i) handgrip strength by a handhold dynamometer, (ii) short-term working memory performance by using error rates and reaction times in the Sternberg task, and (iii) cortical hemodynamics of the prefrontal cortex (PFC) via functional near-infrared spectroscopy (fNIRS).

**Results:**

We observed low to moderate negative correlations (r_p_ =  ~ − 0.38 to − 0.51; p < 0.05) between reaction time and levels of oxyHb in specific parts of the PFC. Furthermore, we noticed low to moderate positive correlations (r_p_ =  ~ 0.34 to 0.45; p < 0.05) between reaction times and levels of deoxyHb in distinct parts of the PFC. Additionally, higher levels of oxyHb (r_p_ (35) = 0.401; p = 0.014) and lower levels of deoxyHb (r_p_ (34) = − 0.338; p = 0.043) in specific parts of the PFC were linked to higher percentage of correct answers. We also found low to moderate correlations (p < 0.05) between measures of handgrip strength and levels of oxyHb (r_p_ =  ~ 0.35; p < 0.05) and levels of deoxyHb (r_p_ =  ~ − 0.25 to − 0.49; p < 0.05) in specific parts of the PFC. However, there was neither a correlation between cognitive performance and handgrip strength nor did cortical hemodynamics in the PFC mediate the relationship between handgrip strength and cognitive performance (p > 0.05).

**Conclusion:**

The present study provides evidence for a positive neurobehavioral relationship between cortical hemodynamics and cognitive performance. Our findings further imply that in younger adults higher levels of handgrip strength positively influence cortical hemodynamics although the latter did not necessarily culminate in better cognitive performance. Future research should examine whether the present findings can be generalized to other cohorts (e.g., older adults).

## Background

Recent reviews provide evidence that the preservation of muscular strength (e.g., due to regular resistance training) is beneficial to maintain brain health and cognitive functioning [1–4].

In this context, handgrip strength has been considered an important marker of health in general [5–8] and of brain health in particular [9–11]. In line with this assumption, there is accumulating evidence linking measures of handgrip strength to cognitive functioning across the human lifespan. More specifically, it has been observed that in older individuals, higher levels of handgrip strength are associated with lesser cognitive decline during aging [12–19] and with better performance in standardized cognitive tests [20–25]. Moreover, also in younger adults [26] and middle-aged adults [27] higher levels of handgrip strength were linked to higher cognitive performance. Accordingly, these findings suggest that even in younger and middle-aged adults, a certain level of (handgrip) strength is an important factor contributing to cognitive well-functioning. However, based on the low number of available studies incorporating those age groups, additional, arguably more critical investigations, are required before strong conclusions can be drawn with certainty.

Notably, none of the mentioned studies help answering the question why higher levels of handgrip strength are linked to better cognitive performance as those studies did not assess the neurophysiological correlates (e.g., cortical hemodynamics). These neurophysiological correlates (e.g., cortical hemodynamics) might mediate the relationship between handgrip strength and cognitive performance [1, 18]. The assumption that changes in cortical hemodynamics (e.g., changes in oxyHb and deoxyHb) can mediate the relationship between handgrip strength and cognitive performance emerged from studies investigating the relationships between cardiorespiratory fitness, cognitive performance and cortical hemodynamics.

In particular, these previous studies observed (i) that higher levels of cardiorespiratory fitness (e.g., objectified by maximal oxygen uptake [${\text{VO}}_{{2_{\max .} }}$]) are associated with better cognitive performance and higher levels of oxyHb in the PFC [28–31] as well as (ii) that cortical hemodynamics (e.g., level of oxyHb concentration) mediate, at least partly, the relationship between cardiorespiratory fitness level and cognitive performance [30–32].

To the best of our knowledge, there is currently no comparable study available that has examined the relationship between muscular strength, cognitive functioning, and cortical hemodynamics [1]. Hence, the current study aims to investigate the possible links between muscular strength (i.e., operationalized by handgrip strength), cognitive functioning (i.e., assessed by reaction times and errors in Sternberg task), and functional cortical hemodynamics (i.e., measured by fNIRS) in younger adults.

## Materials and methods

### Participants and study design

Thirty-nine healthy right-handed, young adults [13 female/26 male; age 24.0 ± 3.1 years; body height 174.4 ± 9.2 cm; body mass 72.7 ± 14.2 kg; body mass index (BMI) 23.7 ± 3.3 kg/m^2^] with normal or corrected vision who had no history of self-reported orthopaedic, cardiovascular, psychiatric, and/or neurological diseases participated in this study.

The study was approved by the local ethics committee of the Medical Faculty of the Otto von Guericke University Magdeburg (No. 181/18) and was in accordance with the Declaration of Helsinki (1964). All participants were informed about the study procedures and provided written informed consent to participate. The participants received a compensation fee of 16 EUR.

In this cross-sectional study, the participants were asked to visit our laboratory once to assess their general participants’ characteristics, to complete questionnaires on mental health, sleep quality, and regular physical activity level, to conduct selected neuropsychological tests, and to assess their handedness and maximal isometric handgrip strength. Furthermore, fNIRS was used to record the cortical hemodynamics while the participants solved the Sternberg task. All tests are described below in more detail.

### Screening measures and handgrip strength testing

The mental health status was assessed by using the Becks Depression Inventory II (BDI-II) in which higher total scores indicate more serve depressive symptoms [33]. Furthermore, we evaluated sleep quality by using the Pittsburgh Sleep Quality Index (PSQI) [34]. A higher total PSQI score indicates more impaired sleep quality [34].

The regular physical activity level and the regular physical exercise level was objectified by using a German-language questionnaire [‘Bewegungs- und Sportsaktivitätsfragebogen’ (BSA-F)] [35]. To rate the level of regular physical activity and the regular physical exercise, the frequency and duration for each activity or exercise needed to be stated and was added up to a single outcome value (in minutes per week).

The time to complete both parts of the Trail-Making Test (TMT A&B) was used to probe the individual performance in visual search (TMT A) and cognitive flexibility (TMT B) [36, 37]. In addition, we calculate the time difference (TMT B-A) between TMT-B and TMT-A as measure of shifting ability [38].

To determine the handedness of the participant, the Edinburgh Handedness Inventory (EHI) [39] with a cut-off value of ± 50 was used (left hander: < − 50; ambidexter: between ≥ − 50 and ≤ + 50; right hander: > + 50) [40].

Handgrip strength was measured using a handhold dynamometer (DHD-1; Saehan®, South Korea) and following the recommendations provided in the Southampton protocol [41]. Accordingly, the participants were seated in a comfortable chair with their feet flat on the ground. The shoulder of the tested extremity was adducted and neutrally rotated. We advised the participants to flex the elbow of the tested extremity at 90° and maintain a neutral wrist position (i.e., thumb facing upward). The participants were asked to squeeze the hand as hard as they can for 3 s. Each participant performed three trials for each hand and after performing one trial, the hand was changed [41]. The maximal handgrip strength of the three trials of each extremity side was used for further analysis. To account for the influence of body composition, we normalized the maximal handgrip strength to the body mass index (BMI) of the participants by using following equation: normalized handgrip strength (NGS) = absolute handgrip strength (in kg)/BMI (in kg/m^2^) [42, 43].

### Cognitive testing

**Fig. 1:**
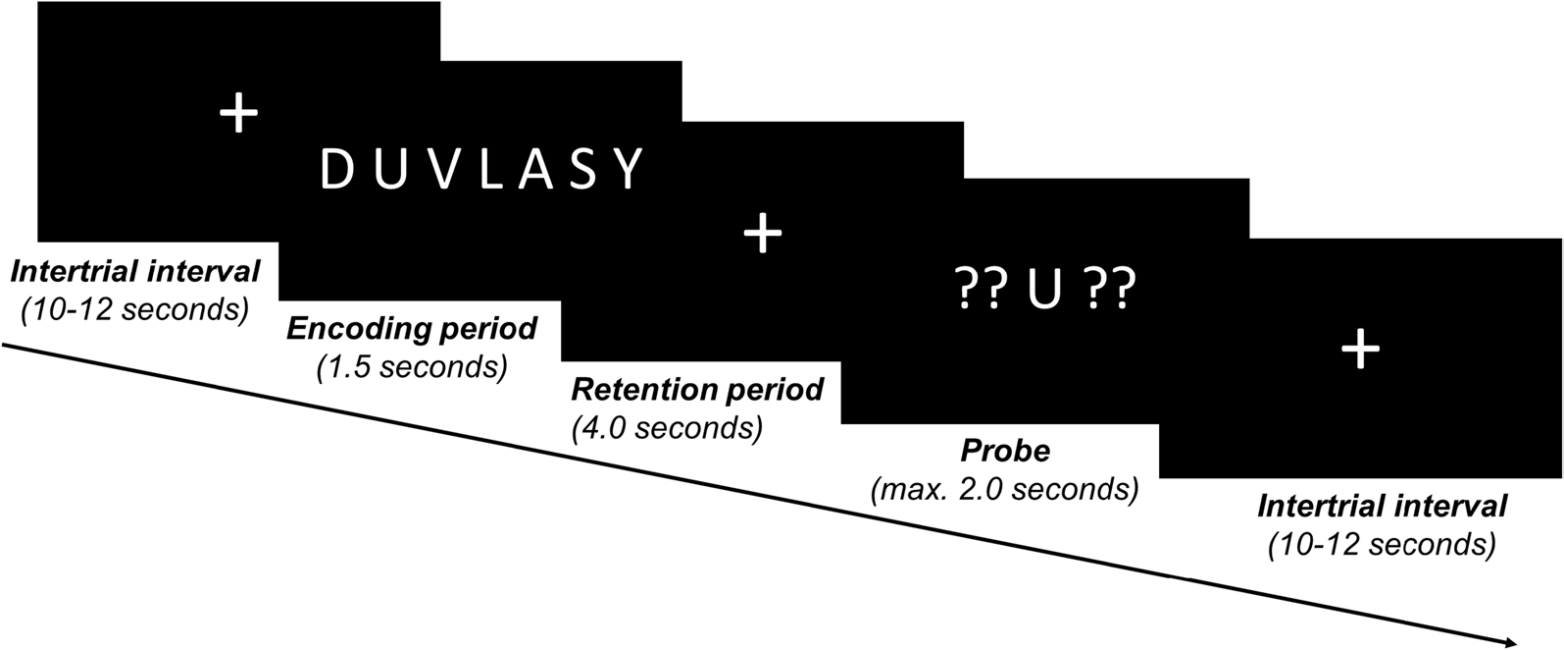
Schematic illustration of a trial of the modified Sternberg task. The trial started with a white fixation cross at the middle of a black screen (first picture: “ + ”) that was presented for a randomly varying period of time between 10 and 12 s. The fixation cross was followed by an encoding period which lasted for 1.5 s. During this period participants had to encode a string of seven letters (second picture: “DUVLASY”). After, a retention period of 4.0 s (third picture: “ + ”) a single probe letter flanked by two questions marks on each side was shown for a maximum duration of 2.0 s (fourth picture: “?? U ??”). At this point, participants were instructed to decide as fast as possible (within the response window of 2 s) whether the probe letter had been included in the encoding period or not. Thereafter, the next trial started with an intertrial interval of 10–12 s (fifth picture: “ + ”)

The Sternberg task which assesses short-term working memory [44] was used in this study because previous publications showed that Sternberg task induces a robust activation of the prefrontal cortex [45–50]. At the beginning of the experiment, each participant was placed in a self-selected comfortable distance to the presentation screen (mean distance to the presentation screen: 67.14 cm; standard deviation: 9.86 cm). We used PsychoPy 2 to present the Sternberg task [51–53]. As shown in Fig. 1, at the beginning of each trial an array of seven letters (i.e., the target memory set) occurred for 1.5 s on the screen and was followed by a retention period with a white fixation cross for 4 s. Afterwards, a probe letter which was flanked by two interrogation marks was shown for no more than 2 s. We asked the subjects to maintain the target memory set over the retention period in mind. When the probe letter occurred, the participants were advised to press the right cursor button (if the presented letter was included in the target memory set) or the left button (if the presented letter was not included in the target memory set) as quickly and accurately as possible.

All participants used the index finger of the right hand to press the left cursor button and middle finger of the right hand to press the right cursor button. Furthermore, after a trial a rest period with a randomized time interval of 10 to 12 s, in which the participants looked at white fix a fixation cross, was included to diminish possible resonance effects [45, 54]. Each participant solved 24 trials and in the half of the trials the target memory set included the probe letter.

During Sternberg task, the behavioral performance for each trial (accuracy and reaction time) and cortical hemodynamics using functional near-infrared spectroscopy were recorded. We averaged accuracy and reaction time across all trials. Accuracy was expressed as accuracy score (percentage of correct answers). In addition, each participant was adequately familiarized with the Sternberg task and solved ten familiarization trials before recording cortical hemodynamics (mean accuracy score: 88.38%; standard deviation: 10.68%, mean reaction time: 1.00 s; standard deviation: 0.25 s).

During the entire duration of the Sternberg task, we measured heart rate signal using a heart rate watch and a chest strap (Polar watch V800® and chest strap H10®, Polar Electro Oy, Kempele, Finland). The heart rate signals were processed using “Kubios HRV” (Biosignal Analysis and Medical Imaging Group, University Kuopio, Finland; Version 3.0.0) [55, 56]. Artefact correction was performed for every participant separately in order to select the optimal threshold for artefact correction [56]. Possible artefacts were removed using a threshold-based filter algorithm which was set to the lowest correction level that cleans all artefacts but does not remove too many normal RR intervals [56]. In the current study, we used a low (35 s) or a medium threshold (0.25 s). Using these thresholds, all values that differ more than 0.25 s (or 0.35 s) from the average value were replaced with interpolated values estimated by a cubic spline interpolation [55–57]. After artefact correction, the HR time series was detrended by applying the smoothness-priors-based detrending approach (smoothing parameter, λ = 500) [55, 56]. For analyzing the frequency bands, the frequency ranges were selected as follows: very low frequency (VLF): 0–0.04 Hz, low frequency (LF): 0.04–0.15 Hz, and high frequency (HF): 0.15–0.4 Hz [55, 56, 58].

### Functional near-infrared spectroscopy

fNIRS is a relatively new, non-invasive neuroimaging technique which is based on theory of neurovascular coupling and optical spectroscopy [54, 59, 60]. fNIRS enables the recording of changes in oxygenated hemoglobin (oxyHb) and deoxygenated hemoglobin (deoxyHb) which allows the “indirect” assessment of cortical activation. A higher cortical activation is commonly indicated by an increase on oxyHb and a decrease in deoxyHb [54, 60]. More detailed information on fNIRS can be found in the referenced literature [54, 59–66].

**Fig. 2:**
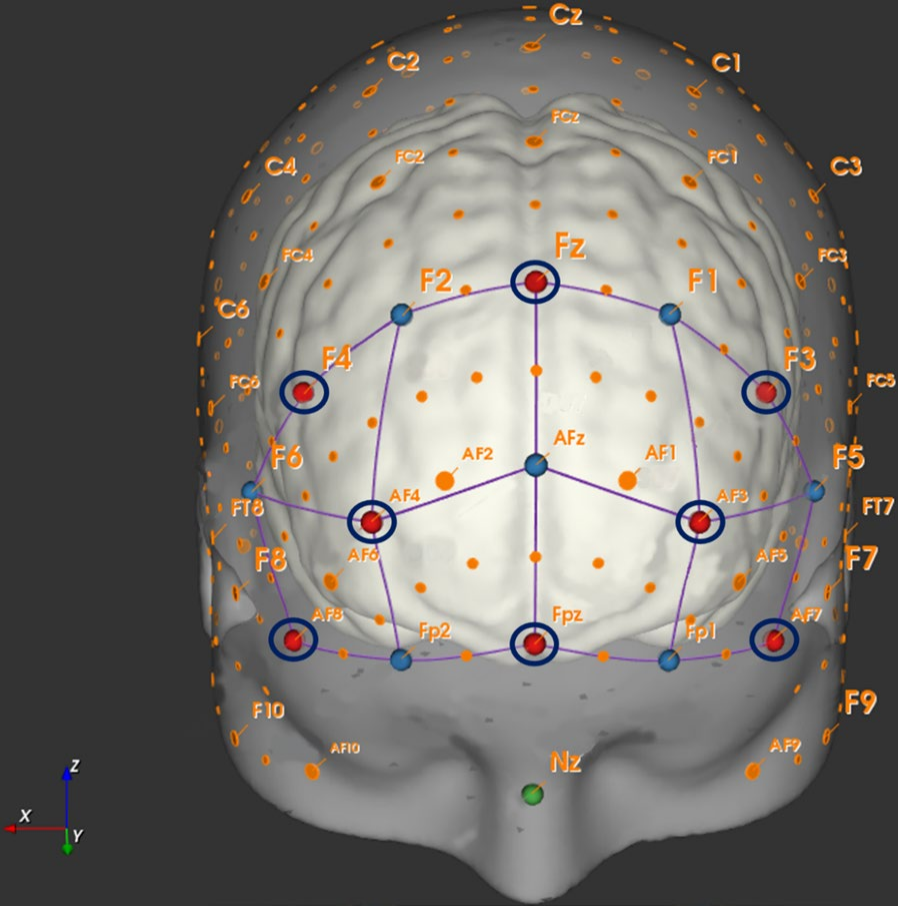
Schematic illustration of the channel setup. The ‘red dots’ represent sources and the ‘blue dots’ represent detectors. A ‘dark blue circle’ around a detector indicates the position of a short-separation channel (SSC; 8 mm). Channels in ‘purple’ are combinations of distinct sources and detectors. The corresponding EEG-positions and the long-separation channel distance (LSC) between sources and detectors result in the following recorded measurement channels: Channel 1 (F4-F2; LSC: 30 mm), Channel 2 (F4-F6; LSC: 30 mm), Channel 3 (AF8-F6; LSC: 33 mm), Channel 4 (AF8-Fp2; LSC: 30 mm), Channel 5 (AF4-F2; LSC: 44 mm), Channel 6 (AF4-F6; LSC: 45 mm), Channel 7 (AF4-Fp2; LSC: 28 mm), Channel 8 (AF4-Afz; LSC: 36 mm), Channel 9 (Fpz-Fp2; LSC: 31 mm), Channel 10 (Fpz-AFz; LSC: 40 mm), Channel 11 (Fpz-Fp1; LSC: 30 mm), Channel 12 (AF3-AFz; LSC: 36 mm), Channel 13 (AF3-Fp1; LSC: 27 mm), Channel 14 (AF3-F5; LSC: 44 mm), Channel 15 (AF3-F1; LSC: 44 mm), Channel 16 (AF7-Fp1; LSC: 30 mm), Channel 17 (AF7-F5; LSC: 33 mm), Channel 18 (F3-F5; LSC: 29 mm), Channel 19 (F3-F1; LSC: 29 mm), Channel 20 (Fz-F2; 29 mm), Channel 21 (Fz-F2; LSC: 40 mm), and Channel 22 (Fz-F1; LSC: 29 mm)

In the current study, we recorded changes in cortical hemodynamics at a frequency of 7.81 Hz by using a portable continuous wave fNIRS system (NIRSport™, NIRx Medical Technologies, Glen Head, New York, USA). Our fNIRS system consists of eight light sources (emitting light at wavelengths of 760 and 850 nm), eight light detectors, and a short-distance detector bundle. The fNIRS optodes were placed according to the 10–20 EEG system [67] using a standardized cap (EasyCap GmbH, Herrsching, Germany). As shown in Fig. 2, our fNIRS setup consists of 22 long source-detector separation channels (LSC; ~ 27 mm to 45 mm) and 8 short source-detector separation channels (SSC; ~ 8 mm). The LSC were used to measure changes in cortical hemodynamics while SSC were used to account for changes in extracerebral blood flow (see “fNIRS data processing”). To assign fNIRS-signals from LSC’s to specific brain regions, we performed a virtual and probabilistic spatial registration using the software fNIRS Optodes’ Location Decider (fOLD) [68] and the Broadmann atlas (BA) [69]. Based on this probabilistic spatial registration, our setup covered the following cortical regions: right dorsolateral prefrontal cortex (BA 9; Channel 1, 5, and 20), right dorsolateral prefrontal cortex (BA 46; Channel 6), left dorsolateral prefrontal cortex (BA 9; Channel 15, 19, and 22), left dorsolateral prefrontal cortex (BA46; Channel 14), right frontopolar area (BA 10; Channel 4, 7, 8, and 9), left frontopolar area (BA 10; Channel 11, 12, 13, and 16), right pars triangularis of Broca’s area (BA 45; Channel 2 and 3), left pars triangularis of Broca’s area (BA 45; Channel 17 and 18), middle frontopolar area (BA 10; Channel 10), and middle dorsolateral prefrontal cortex (BA 9; Channel 21). More detailed information on spatial registration is provided in Additional file 1: Table S1.

### fNIRS data processing

The fNIRS data were preprocessed using the software program Homer 2 (version 2.8) [70] and followed current data processing recommendations [54, 71]. At first, we used enPruneChannels function with a signal-to-noise threshold (SNR thres) of 10 to exclude to noisy channels [coefficient of variation (CV) > 10] from further analyses. In this study 1.5% of channels were excluded because they were considered as to noisy which were on average 0.36 channels per individual. Then, the raw light intensity signals were converted into changes in optical density (using hmrIntensity2OD function) [70]. Afterwards, the fNIRS time series were corrected for motion artefacts [59, 72]. For this purpose, we used the hmrMotionCorrectWavelet filtering function implemented in Homer 2 which is based on the algorithm described by Molavi and Dumont [73]. The threshold of the wavelet filter was set to 1.219 times of interquartile [74–76] that corresponds to the recommend α of 0.1 [72, 73, 77]. Following the motion artefact correction, we used a bandpass filter (hmrBandpassFilt function) with a high-pass cut-off frequency of 0.01 Hz to account for instrumental noise and low frequency drifts and a low-pass cut-off frequency of 0.09 Hz to account for cardiac oscillations and Mayer-waves [71]. Subsequently, preprocessed optical density data of both wavelengths were converted via the modified Beer–Lambert law (MBLL) into concentration changes of oxygenated hemoglobin (oxyHb) and deoxygenated hemoglobin (deoxyHb) by using the hmrOD2Conc function. The differential pathlength factor which is required in the MBLL was calculated for each individual participant using the equation provided by Scholkmann and Wolf [78].

Afterwards, we used the hmrDeconvHRRF_DriftSS function to account for the confounding effects of extracerebral (superficial) blood flow. This function is based on the assumptions that SSC record changes in extracerebral blood flow whereas LSC measures both change in superficial blood flow and cortical brain tissue [54, 79]. Hence, the signals of SSC can be used to regress out signals from extracerebral layers from LSC which result in an improved data quality and help to avoid “false positives” [80]. In the hmrDeconvHRRF_DriftSS function, the hemodynamic response function (HRF) is estimated by using a general linear model approach (GLM) that uses ordinary least squares [81]. As done in several previous publications [82–90], the HRF was modeled with a consecutive sequence of Gaussian functions with a standard deviation of 0.5 s and their means separated by 0.5 s over a specific regression time (used parameters in Homer 2: trange − 2.0 to 20; glmSolveMethod 1; idxBasis 1; paramsBasis 0.5 and 0.5). To account for baseline drift, it was modeled using a third order polynomial fit [84, 89, 91]. Furthermore, we used the nearest SSC as static estimator for regression because the location of the SSC impacts the performance of the regression analysis substantially [92]. Following the SSC regression, we used the hmrBlockAvg function to perform a baseline correction and to calculate the block averages for oxyHb and deoxyHb changes over all trials and for each measurement channel (i.e., each LSC). In order to baseline correct our data, we used a time period of 2 s prior to stimulus onset which is a commonly utilized time period in event-related fNIRS studies (for review see [54]). The entire period of the stimulus phase was used to calculate baseline-corrected block averages (i.e., 0 to 20 s after stimulus onset).

After pre-processing with Homer 2, the baseline-corrected block averages of the time series of oxyHb and deoxyHb were exported and imported into Matlab (The Mathworks®, Natick, Massachusetts, USA). Using the exported block averages and an in-house Matlab software, we calculated the median values of oxyHb and deoxyHb for each fNIRS measurement channel across a period of 0 to 16 s after trial onset. Median values were used for further statistical analyses because they are deemed to be less influenced by potential outliers [54, 59].

### Statistical analysis

The statistical analysis was performed using IBM SPSS (Statistical Package for Social Science, Version 26, Chicago, Illinois, USA). To investigate whether cortical hemodynamics (e.g., oxyHb and deoxyHb) mediate the relationship between measures of handgrip strength and measures of cognitive performance (i.e., reaction time in Sternberg task), a mediation analysis was conducted. Thereto, we assessed in the first step, normal distribution using the Shapiro–Wilk test. As the most parameters (especially fNIRS parameters) were not normally distributed, we used non-parametric methods in the subsequent statistical analysis. In the second step, we examined the bivariate relationships (i) between measures of handgrip strength and measures of cognitive performance, (ii) between measures of cognitive performance and measures of cortical hemodynamics, and (iii) between measures of cortical hemodynamics and measures of handgrip strength by calculating non-parametric partial correlations (r_p_) controlling for age and gender [93, 94]. The correlations were rated as follows: 0 to 0.19: no correlation; 0.2 to 0.39: low correlation, 0.40 to 0.59: moderate correlation; 0.60 to 0.79: moderately high correlation; ≥ 0.80: high correlation [95, 96]. The level of statistical significance was set to α = 0.05 in correlation analysis.

In the third step, we performed a robust mediation analysis using the SPSS extension bundle “robmed” [97] and the calculated mediation model includes a three-output analyses process (Paths A–C′). In this regard, the associations between (i) the independent variable (measures of handgrip strength) and dependent variable (measures of cognitive performance)—Path C, (ii) the independent variable (measures of handgrip strength) and the mediator (measures of cortical hemodynamics)—Path A, and (iii) the mediator (measures of cortical hemodynamics) and the dependent variable (measures of cognitive performance)—Path B, were computed. Afterwards, the direct effect (Path C′) of the independent variable (e.g., measures of handgrip strength) on the dependent variable (e.g., measures of cognitive performance) was estimated by controlling for the mediator (measures of cortical hemodynamics) and the indirect effect (Path AB). To calculate the direct and indirect effects, we computed a robust mediation model [97] with bias-corrected bootstrap 95% confidence intervals (CIs) based on 5000 bootstrap resamples and entered the covariates age and gender [93, 94]. In accordance with the literature, a significant mediation was assumed if the CIs in Path AB did not include zero whereas a partial mediation was indicated if the CIs of Path C′ cross zero [98–100]. Please note a mediation analysis was only computed if two phenomena appeared: firstly, if the non-parametric partial correlation analysis (see “second step” and Tables 2 and 3) exhibited that there was a significant correlation between mediator and independent variable (or dependent variable) and secondly, if there was a correlation of at least r_p_ ≥ 0.2 between mediator and dependent variable (or independent variable). We selected a threshold of r_p_ ≥ 0.2 because this is deemed to be the minimum effect size that represents a “practical” significant effect [101].

## Results

The general characteristics of the participants are shown in Table 1.

**Table 1:** Median and interquartile range of the screening tests in the investigated sample

Parameters	Median ± interquartile range
BDI-II (total score)	3.0 ± 6.0
Level of education (years)	15.6 ± 2.4
PSQI (total score)	4.0 ± 2.0
BSA (min per week)	PA: 236.5 ± 255.0/PE:292.5 ± 350.5
TMT A (s)	19.4 ± 6.3
TMT B (s)	42.9 ± 14.4
TMT B-A (s)	22.9 ± 11.4
EHI (total score)	83.3 ± 17.3
Mean heart rate during ST (bpm)	71.8 ± 20.2
LF/HF ratio	1.43 ± 1.51
Left/Right AHS (kg)	38.9 ± 26.2/44.1 ± 22.7
Left/Right NHS (A.U.)	1.67 ± 0.87/1.87 ± 0.78
Reaction time in ST (s)	0.94 ± 0.37
Correct answers in ST (%)	87.5 ± 12.5

*AHS* absolute handgrip strength, *A.U.* arbitrary unit, *BDI-II* Becks Depression Inventory II (a score of 13 and higher indicates depression [102, 103]), *bpm* beats per minute, *BSA* physical activity questionnaire, derived from German ‘Bewegungs- und Sportaktivitätsfragebogen’ (The World Health Organization recommends, at least, 150 min of moderate-intensity physical activity in a week for substantial health benefits [104]), *EHI* Edinburgh Handedness Inventory (a score of 50 and higher indicates right-handedness [40]), *LF/HF ratio* low-frequency/high-frequency ratio, *kg* kilogram, *min* minutes, *NHS* normalized handgrip strength (normalized to body-mass-index), *PA* physical activity, *PE* physical exercise, *s* seconds, *PSQI* Pittsburgh Sleep Quality Index (a score of 6 and higher indicates insomnia [105]), *ST* Sternberg task, *TMT* Trail Making Test

### Correlations between measures of handgrip strength and cognitive performance in Sternberg task

The non-parametric partial correlations (controlling for the influence of age and gender) between left- and right-hand absolute handgrip strength (AHS) and reaction time in Sternberg task [left AHS: r_p_ (35) = − 0.278; p = 0.096/right AHS: r_p_ (35) = − 0.157; p = 0.354] and between left- and right-hand normalized handgrip strength (NHS) and reaction time in Sternberg task [left NHS: r_p_ (35) = − 0.253; p = 0.131/right NHS: r_p_ (35) = − 0.134; p = 0.431] did not reach statistical significance. Furthermore, we did also not observe statistically significant correlations between left- and right-hand absolute handgrip strength and correct answers in Sternberg task [left AHS: r_p_ (35) = 0.012; p = 0.945/right AHS: r_p_ (35) = − 0.045; p = 0.791] and between left- and right-hand normalized handgrip strength and correct answers in Sternberg task [left NHS: r_p_ (35) = 0.122; p = 0.472/right AHS: r_p_ (35) = 0.091; p = 0.592].

### Correlations between measures of handgrip strength, measures of cognitive performance, and measures of cortical hemodynamics

The results of the partial correlation analysis (controlling for the influence of age and gender) between measures of handgrip strength, cognitive performance, and cortical hemodynamics are shown in Table 2 (oxyHb) and Table 3 (deoxyHb).

**Table 2:** Overview of the regional cortical changes in the concentrations of oxygenated hemoglobin (oxyHb) measured during the Sternberg task (ST) and their correlations with measures of handgrip strength and cognitive performance

Channels	Median ± IQR	Non-parametric partial correlations		
	OxyHb (µmol/L)	AHS (left/right)	NHS (left/right)	Reaction time in ST/correct answers in ST
1 (rt. DLPFC; BA9)	0.017 ± 0.028	r_p_ (35) = 0.179; p = 0.288/r_p_ (35) = 0.241; p = 0.151	r_p_ (35) = 0.145; p = 0.391/r_p_ (35) = 0.190; p = 0.260	*r*_*p*_* (35)* = − *0.417; p* = *0.010/r*_*p*_* (35)* = *0.401; p* = *0.014*
2 (rt. Broca; BA 45)	0.013 ± 0.058	r_p_ (35) = 0.287; p = 0.085/r_p_ (35) = 0.161; p = 0.340	r_p_ (35) = 0.210; p = 0.212/r_p_ (35) = 0.134; p = 0.430	*r*_*p*_* (35)* = − *0.426; p* = *0.009*/r_p_ (35) = 0.206; p = 0.222
3 (rt. Broca; BA 45)	0.017 ± 0.053	r_p_ (34) = 0.021; p = 0.905/r_p_ (34) = 0.075; p = 0.663	r_p_ (34) = 0.036; p = 0.833/r_p_ (34) = 0.108; p = 0.530	*r*_*p*_* (34)* = − *0.388; p* = *0.019*/r_p_ (34) = 0.241; p = 0.156
4 (rt. FPA; BA 10)	− 0.018 ± 0.073	r_p_ (35) = − 0.021; p = 0.903/r_p_ (35) = − 0.007; p = 0.968	r_p_ (35) = − 0.094; p = 0.581/r_p_ (35) = − 0.061; p = 0.719	r_p_ (35) = − 0.170; p = 0.316/r_p_ (35) = 0.249; p = 0.138
5 (rt. DLPFC; BA 9)	0.016 ± 0.052	*r*_*p*_* (32)* = *0.359; p* = *0.037*/r_p_ (32) = 0.257; p = 0.143	r_p_ (32) = 0.225; p = 0.202/r_p_ (32) = 0.147; p = 0.407	*r*_*p*_* (32)* = − *0.404; p* = *0.018*/r_p_ (32) = 0.177; p = 0.317
6 (rt. DLPFC; BA 46)	0.021 ± 0.077	r_p_ (34) = 0.157; p = 0.360/r_p_ (34) = 0.174; p = 0.310	r_p_ (34) = 0.087; p = 0.613/r_p_ (34) = 0.161; p = 0.347	*r*_*p*_* (34)* = − *0.431; p* = *0.009*/r_p_ (34) = 0.227; p = 0.182
7 (rt. FPA; BA 10)	0.012 ± 0.035	r_p_ (34) = 0.005; p = 0.979/r_p_ (34) = 0.053; p = 0.759	r_p_ (34) = − 0.068; p = 0.692/r_p_ (34) = 0.009; p = 0.958	r_p_ (34) = 0.091; p = 0.598/r_p_ (34) = − 0.160; p = 0.351
8 (rt. FPA; BA 10)	0.008 ± 0.049	r_p_ (35) = 0.142; p = 0.402/r_p_ (35) = 0.128; p = 0.451	r_p_ (35) = 0.013; p = 0.940/r_p_ (35) = 0.008; p = 0.964	r_p_ (35) = − 0.299; p = 0.072/r_p_ (35) = 0.145; p = 0.391
9 (rt. FPA; BA 10)	− 0.037 ± 0.043	r_p_ (35) = 0.135; p = 0.427/r_p_ (35) = 0.108; p = 0.524	r_p_ (35) = 0.120; p = 0.481/r_p_ (35) = 0.116; p = 0.494	r_p_ (35) = − 0.091; p = 0.593/r_p_ (35) = 0.221; p = 0.188
10 (md. FPA; BA 10)	− 0.004 ± 0.037	r_p_ (35) = 0.246; p = 0.143/r_p_ (35) = 0.125; p = 0.460	r_p_ (35) = 0.273; p = 0.102/r_p_ (35) = 0.152; p = 0.370	*r*_*p*_* (35)* = − *0.518; p* = *0.001*/r_p_ (35) = 0.173; p = 0.306
11 (lt. FPA; BA 10)	− 0.019 ± 0.056	r_p_ (35) = 0.102; p = 0.546/r_p_ (35) = − 0.018; p = 0.915	r_p_ (35) = 0.093; p = 0.585/r_p_ (35) = − 0.014; p = 0.934	*r*_*p*_* (35)* = − *0.416; p* = *0.010*/r_p_ (35) = 0.045; p = 0.793
12 (lt. FPA; BA 10)	0.004 ± 0.049	r_p_ (35) = 0.295; p = 0.077/r_p_ (35) = 0.141; p = 0.405	r_p_ (35) = 0.214; p = 0.205/r_p_ (35) = 0.072; p = 0.672	*r*_*p*_* (35)* = − *0.393; p* = *0.016*/r_p_ (35) = 0.103; p = 0.545
13 (lt. FPA; BA 10)	− 0.003 ± 0.052	r_p_ (34) = 0.244; p = 0.152/r_p_ (34) = 0.102; p = 0.555	r_p_ (34) = 0.152; p = 0.375/r_p_ (34) = 0.017; p = 0.923	r_p_ (35) = − 0.099; p = 0.566/r_p_ (35) = − 0.088; p = 0.610
14 (lt. DLPFC; BA 46)	0.018 ± 0.057	r_p_ (34) = 0.088; p = 0.609/r_p_ (34) = 0.066; p = 0.704	r_p_ (34) = 0.048; p = 0.782/r_p_ (34) = 0.088; p = 0.611	r_p_ (34) = − 0.118; p = 0.492/r_p_ (34) = 0.207; p = 0.225
15 (lt. DLPFC; BA 9)	0.002 ± 0.037	r_p_ (32) = 0.269; p = 0.125/r_p_ (32) = 0.095; p = 0.593	r_p_ (32) = 0.242; p = 0.169/r_p_ (32) = 0.060; p = 0.736	*r*_*p*_* (32)* = − *0.459; p* = *0.006*/r_p_ (32) = 0.202; p = 0.251
16 (lt. FPA; BA 10)	− 0.008 ± 0.063	r_p_ (35) = 0.115; p = 0.498/r_p_ (35) = 0.167; p = 0.322	r_p_ (35) = 0.115; p = 0.498/r_p_ (35) = 0.171; p = 0.311	r_p_ (35) = 0.000; p = 1.000/r_p_ (35) = 0.122; p = 0.474
17 (lt. Broca; BA 45)	0.019 ± 0.071	r_p_ (35) = 0.165; p = 0.329/r_p_ (35) = 0.116; p = 0.493	r_p_ (35) = 0.165; p = 0.329/r_p_ (35) = 0.173; p = 0.306	r_p_ (35) = − 0.230; p = 0.170/r_p_ (35) = 0.133; p = 0.431
18 (lt. Broca; BA 45)	0.013 ± 0.051	r_p_ (35) = 0.089; p = 0.601/r_p_ (35) = 0.030; p = 0.860	r_p_ (35) = − 0.014; p = 0.936/r_p_ (35) = 0.007; p = 0.970	r_p_ (35) = 0.017; p = 0.922/r_p_ (35) = 0.033; p = 0.844
19 (lt. DLPFC; BA 9)	0.014 ± 0.035	r_p_ (34) = 0.109; p = 0.527/r_p_ (34) = 0.020; p = 0.908	r_p_ (34) = 0.021; p = 0.902/r_p_ (34) = − 0.029; p = 0.868	r_p_ (34) = − 0.131; p = 0.445/r_p_ (34) = 0.089; p = 0.606
20 (rt. DLPFC; BA 9)	0.009 ± 0.031	r_p_ (35) = 0.241; p = 0.151/r_p_ (35) = 0.288; p = 0.084	r_p_ (35) = 0.154; p = 0.362/r_p_ (35) = 0.243; p = 0.147	r_p_ (35) = − 0.042; p = 0.806/r_p_ (35) = 0.234; p = 0.164
21 (md. DLPFC; BA 9)	0.003 ± 0.032	*r*_*p*_* (34)* = *0. 358; p* = *0.032*/r_p_ (34) = 0.230; p = 0.178	r_p_ (34) = 0.228; p = 0.181/r_p_ (34) = 0.088; p = 0.612	r_p_ (34) = − 0.315; p = 0.061/r_p_ (34) = 0.299; p = 0.076
22 (lt. DLPFC; BA 9)	0.001 ± 0.029	r_p_ (35) = 0.110; p = 0.517/r_p_ (35) = − 0.030; p = 0.860	r_p_ (35) = 0.092; p = 0.587/r_p_ (35) = − 0.054; p = 0.752	*r*_*p*_* (35)* = − *0.389; p* = *0.017*/r_p_ (35) = 0.248; p = 0.139

On the left hand of the table, median ± interquartile range (IQR) of concentrations of oxyHb are displayed. On the right hand of the table, non-parametric correlations (controlled for age and gender) between oxyHb and absolute handgrip strength (AHS), normalized handgrip strength (NHS), reaction time and percentage of correct answers in the ST are shown. Significant correlations are presented in italic

*BA* broadman area, *DLPFC* dorsolateral prefrontal cortex, *FPA* frontopolar area, *lt.* left, *md.* middle, *r*_*p*_ partial correlation controlling for age and gender, *rt.* right, *ST* Sternberg task

**Table 3:** Overview of the regional cortical changes in the concentrations of deoxygenated hemoglobin (deoxyHb) measured during the Sternberg task (ST) and their correlations with measures of handgrip strength and cognitive performance

Channels	Median ± IQR	Non-parametric partial correlations		
	DeoxyHb (µmol/L)	AHS (left/right)	NHS (left/right)	Reaction time in ST/correct answers in ST
1 (rt. DLPFC; BA9)	0.001 ± 0.005	r_p_ (35) = − 0.197; p = 0.243/r_p_ (35) = − 0.182; p = 0.280	r_p_ (35) = − 0.232; p = 0.167/r_p_ (35) = − 0.245; p = 0.144	r_p_ (35) = 0.198; p = 0.240/r_p_ (35) = − 0.276; p = 0.099
2 (rt. Broca; BA 45)	0.000 ± 0.015	r_p_ (35) = − 0.299; p = 0.072/r_p_ (35) = − 0.180; p = 0.286	r_p_ (35) = − 0.204; p = 0.225/r_p_ (35) = − 0.133; p = 0.434	*r*_*p*_* (35)* = *0.343; p* = *0.038*/r_p_ (35) = − 0.142; p = 0.401
3 (rt. Broca; BA 45)	− 0.002 ± 0.011	r_s_ (34) = − 0.071; p = 0.680/r_p_ (34) = − 0.203; p = 0.234	r_p_ (34) = − 0.088; p = 0.609/r_p_ (34) = − 0.227; p = 0.183	r_p_ (34) = 0.262; p = 0.122/r_p_ (35) = − 0.199; p = 0.245
4 (rt. FPA; BA 10)	0.000 ± 0.023	r_p_ (35) = − 0.301; p = 0.070/r_p_ (35) = − 0.191; p = 0.258	r_p_ (35) = − 0.294; p = 0.077/r_p_ (35) = − 0.224; p = 0.182	r_p_ (34) = 0.172; p = 0.307/r_p_ (35) = − 0.095; p = 0.574
5 (rt. DLPFC; BA 9)	0.000 ± 0.015	*r*_*p*_* (32)* = − *0.442; p* = *0.009/r*_*p*_* (32)* = − *0.385; p* = *0.025*	*r*_*p*_* (32)* = − *0.454; p* = *0.007/r*_*p*_* (32)* = − *0.390; p* = *0.023*	*r*_*p*_* (32)* = *0.441; p* = *0.009*/r_p_ (32) = − 0.236; p = 0.179
6 (rt. DLPFC; BA 46)	− 0.006 ± 0.019	r_p_ (34) = − 0.040; p = 0.818/r_p_ (34) = − 0.112; p = 0.514	r_p_ (34) = 0.005; p = 0.977/r_p_ (34) = − 0.088; p = 0.610	*r*_*p*_* (34)* = *0.459; p* = *0.005*/r_p_ (34) = − 0.275; p = 0.105
7 (rt. FPA; BA 10)	0.003 ± 0.012	*r*_*p*_* (34)* = − *0.379; p* = *0.023/r*_*p*_* (34)* = − *0.420; p* = *0.011*	*r*_*p*_* (34)* = − *0.339; p* = *0.043/r*_*p*_* (34)* = − *0.368; p* = *0.027*	r_p_ (34) = 0.306; p = 0.070/r_p_ (34) = − 0.177; p = 0.300
8 (rt. FPA; BA 10)	0.002 ± 0.013	*r*_*p*_* (35)* = − *0.363; p* = *0.027/r*_*p*_* (35)* = − *0.429; p* = *0.008*	*r*_*p*_ *(35) = − 0.347; p = 0.035/r*_*p*_ *(35) = − 0.434; p = 0.007*	r_p_ (35) = 0.150; p = 0.376/r_p_ (35) = − 0.113; p = 0.504
9 (rt. FPA; BA 10)	0.002 ± 0.014	r_p_ (35) = − 0.200; p = 0.234/r_p_ (35) = − 0.199; p = 0.239	r_p_ (35) = − 0.252; p = 0.133/r_p_ (35) = − 0.218; p = 0.194	r_p_ (35) = 0.244; p = 0.145/r_p_ (35) = − 0.113; p = 0.507
10 (md. FPA; BA 10)	0.001 ± 0.009	*r*_*p*_ *(35) = − 0.340; p = 0.039/*r_p_ (35) = − 0.306; p = 0.066	r_p_ (35) = − 0.213; p = 0.205/r_p_ (35) = − 0.188 p = 0.265	r_p_ (35) = 0.031; p = 0.856/r_p_ (35) = 0.047; p = 0.781
11 (lt. FPA; BA 10)	0.002 ± 0.012	r_p_ (35) = − 0.194; p = 0.251/r_p_ (35) = − 0.132; p = 0.437	r_p_ (35) = − 0.072; p = 0.670/r_p_ (35) = − 0.021; p = 0.904	r_p_ (35) = 0.164; p = 0.331/r_p_ (35) = − 0.085; p = 0.618
12 (lt. FPA; BA 10)	0.002 ± 0.010	*r*_*p*_* (35)* = − *0.390; p* = *0.017*/r_p_ (35) = − 0.264; p = 0.115	r_p_ (35) = − 0.263; p = 0.116/r_p_ (35) = − 0.131; p = 0.438	r_p_ (35) = 0.147; p = 0.385/r_p_ (35) = 0.048; p = 0.776
13 (lt. FPA; BA 10)	0.002 ± 0.011	*r*_*p*_* (34)* = − *0.436; p* = *0.008*/r_p_ (34) = − 0.326; p = 0.052	*r*_*p*_* (34)* = − *0.396; p* = 0.017/r_p_ (34) = − 0.252; p = 0.137	r_p_ (34) = − 0.163; p = 0.341/r_p_ (34) = 0.018; p = 0.916
14 (lt. DLPFC; BA 46)	− 0.003 ± 0.025	r_p_ (34) = − 0.263; p = 0.122/r_p_ (34) = − 0.270; p = 0.112	r_p_ (34) = − 0.240; p = 0.159/r_p_ (34) = − 0.312; p = 0.064	r_p_ (34) = 0.101; p = 0.557/*r*_*p*_* (34)* = − *0.338; p* = *0.043*
15 (lt. DLPFC; BA 9)	0.002 ± 0.017	*r*_*p*_* (32)* = − *0.409; p* = *0.016*/r_p_ (32) = − 0.326; p = 0.060	*r*_*p*_* (32)* = − *0.493; p* = *0.003*/*r*_*p*_* (32)* = − *0.411; p* = *0.016*	r_p_ (32) = 0.127; p = 0.474/r_p_ (32) = − 0.027; p = 0.879
16 (lt. FPA; BA 10)	0.001 ± 0.021	r_p_ (35) = − 0.206; p = 0.222/r_p_ (35) = − 0.207; p = 0.219	r_p_ (35) = − 0.097; p = 0.570/r_p_ (35) = − 0.073; p = 0.667	r_p_ (35) = − 0.027; p = 0.872/r_p_ (35) = 0.003; p = 0.986
17 (lt. Broca; BA 45)	− 0.003 ± 0.017	r_p_ (35) = − 0.151; p = 0.373/r_p_ (35) = − 0.210; p = 0.213	r_p_ (35) = − 0.188; p = 0.264/r_p_ (35) = − 0.244; p = 0.145	r_p_ (35) = 0.020; p = 0.905/r_p_ (35) = − 0.021; p = 0.901
18 (lt. Broca; BA 45)	0.002 ± 0.009	r_p_ (35) = 0.281; p = 0.092/r_p_ (35) = 0.296; p = 0.075	r_p_ (35) = 0.293; p = 0.078/r_p_ (35) = 0.263; p = 0.116	r_p_ (35) = − 0.055; p = 0.746/r_p_ (35) = − 0.130; p = 0.445
19 (lt. DLPFC; BA 9)	0.001 ± 0.008	r_p_ (34) = − 0.123; p = 0.474/r_p_ (34) = − 0.181; p = 0.291	r_p_ (34) = − 0.080; p = 0.645/r_p_ (34) = − 0.168; p = 0.327	r_p_ (34) = − 0.154; p = 0.371/r_p_ (34) = 0.067; p = 0.698
20 (rt. DLPFC; BA 9)	0.000 ± 0.011	r_p_ (35) = − 0.226; p = 0.179/r_p_ (35) = − 0.277; p = 0.097	r_p_ (35) = − 0.185; p = 0.272/*r*_*p*_* (35)* = − *0.328; p* = *0.048*	r_p_ (35) = − 0.053; p = 0.756/r_p_ (35) = − 0.022; p = 0.898
21 (md. DLPFC; BA 9)	0.003 ± 0.010	r_p_ (34) = − 0.276; p = 0.103/r_p_ (34) = − 0.230; p = 0.178	r_p_ (34) = − 0.315; p = 0.061/r_p_ (34) = − 0.325; p = 0.053	r_p_ (34) = − 0.029; p = 0.869/r_p_ (34) = − 0.113; p = 0.511
22 (lt. DLPFC; BA 9)	0.002 ± 0.009	r_p_ (35) = − 0.155; p = 0.359/r_p_ (35) = − 0.085; p = 0.618	r_p_ (35) = − 0.144; p = 0.395/r_p_ (35) = − 0.103; p = 0.545	r_p_ (35) = − 0.073; p = 0.666/r_p_ (35) = 0.003; p = 0.984

On the left hand of the table, median ± interquartile range (IQR) of concentrations of deoxyHb are displayed. On the right hand of the table, non-parametric partial correlations (controlled for age and gender) between deoxyHb and absolute handgrip strength (AHS), normalized handgrip strength (NHS), reaction time and percentage of correct answers in the ST are shown. Significant correlations are presented in italic

*BA* broadman area, *DLPFC* dorsolateral prefrontal cortex, *FPA* frontopolar area, *lt.* left, *md.* middle, *r*_*p*_ partial correlation controlling for the influence of age and gender, *rt.* right, *ST* Sternberg task

With regard to oxyHb, we observed a low positive correlation between the level of oxyHb in right dorsolateral prefrontal cortex (DLPFC) and middle DLPFC (Ch. 5 and Ch. 21) and left AHS. Furthermore, we found moderate negative correlations between level of oxyHb in specific channels of frontopolar area (FPA) and DLPFC and reaction time, and a moderate positive correlation between level of oxyHb in right DLPFC (Channel 1) and percentage of correct answers in Sternberg task (for a detailed overview see Table 2). Hence, better cognitive performance (e.g., faster reaction time and higher percentage of correct answers) is linked to higher levels of oxyHb (indicating, in general, a higher cortical activation) in distinct parts of the PFC.

With regard to deoxyHb, we observed low to moderate positive correlations between the level of deoxyHb in right FPA and left and right DLPFC and AHS and NHS (for a detailed overview see Table 3). Furthermore, we found a moderate positive correlation between level of deoxyHb in right Borca area (Channel 2) and right DLPFC (Channel 5 and 6) and reaction time in Sternberg task. In addition, we noticed a low and negative correlation between the level of deoxyHb in left DLPFC (Channel 14) and percentage of correct answers in Sternberg task. Collectively, better cognitive performance (e.g., faster reaction time and higher percentage of correct answers) is associated with lower levels of deoxyHb (indicating, in general, a higher cortical activation) in distinct parts of the PFC.

### Mediation analysis

The results of the mediation analysis for the channels which meet our criteria to conduct mediation analysis (see ‘Statistical analysis’) are shown in Table 4.

**Table 4:** Mediation models

Independent variable (x)/mediator (m)/dependent variable (y)	Path A	Path B	Path AB (indirect effect)	Path C (direct effect)
oxyHb				
Right AHS/Ch. 1/CA in ST	β = 0.000 (0.000); z (39) = 1.828; p = 0.068	β = 64.051 (52.713); z (39) = 1.498; p = 0.134	β = 0.025; CI [− 0.008 to 0.095]	β = − 0.003 (0.075); z (39) = − 0.234; p = 0.815
Left AHS/Ch. 2/RT in ST	*β* = *0.001 (0.000);* *z (39) = 2.189; p* = *0.029*	*β* = − *1.854 (0.636);* *z (39) = − 2.948; p* = *0.003*	β = − 0.002; CI [− 0.005 to 0.000]	*β* = − *0.004 (0.002); z (39)* = − *2.175; p* = *0.030*
Left NHS/Ch. 2/RT in ST	β = 0.016 (0.011); z (39) = 1.537; p = 0.124	*β* = − *1.954 (0.710);* *z (39) = − 2.801; p* = *0.005*	β = − 0.032; CI [− 0.097 to 0.003]	β = − 0.082 (0.050); z (39) = − 1.862; p = 0.063
Left AHS/Ch. 5/RT in ST	β = 0.001 (0.000); z (36) = 1.694; p = 0.090	*β* = − *3.004 (1.158);* *z (36) = − 2.523; p* = *0.012*	β = − 0.002; CI [− 0.007 to 0.000]	β = − 0.002 (0.003); z (36) = − 0.680; p = 0.497
Left NHS/Ch. 5/RT in ST	β = 0.014 (0.011); z (36) = 1.271; p = 0.204	*β* = − *3.076 (1.056); * *z (36) = − 2.857; p* = *0.004*	β = − 0.042; CI [− 0.150 to 0.012]	β = − 0.038 (0.061); z (36) = − 0.661; p = 0.509
Left AHS/Ch. 10/RT in ST	β = 0.000 (0.000); z (39) = 1.315; p = 0.188	*β* = − *4.091 (1.362);* *z (39) = − 3.008; p* = *0.003*	β = − 0.002; CI [− 0.007 to 0.001]	β = − 0.003 (0.003); z (39) = − 1.230; p = 0.219
Left NHS/Ch. 10/RT in ST	β = 0.010 (0.010); z (39) = 1.237; p = 0.216	*β* = − *4.087 (1.290);* *z (39) = − 3.190; p* = *0.001*	β = − 0.041; CI [− 0.170 to 0.016]	β = − 0.062 (0.062); z (39) = − 0.998; p = 0.318
Left AHS/Ch. 12/RT in ST	β = 0.001 (0.000); z (39) = 1.445; p = 0.148	β = − 1.949 (1.757); z (39) = − 1.213; p = 0.225	β = − 0.001; CI [− 0.005 to 0.001]	β = − 0.004 (0.003); z (39) = − 1.157; p = 0.247
Left NHS/Ch. 12/RT in ST	β = 0.009 (0.009); z (39) = 0.661; p = 0.509	β = − 2.101 (1.747); z (39) = − 1.310; p = 0.190	β = − 0.018; CI [− 0.101 to 0.010]	β = − 0.075 (0.066); z (39) = − 1.211; p = 0.226
Left AHS/Ch. 15/RT in ST	β = 0.001 (0.000); z (36) = 1.557; p = 0.119	β = − 2.227 (1.656); z (36) = − 1.587; p = 0.112	β = − 0.002; CI [− 0.007 to 0.000]	β = − 0.002 (0.003); z (36) = − 0.533; p = 0.594
Left NHS/Ch. 15/RT in ST	β = 0.018 (0.009); z (36) = 1.761; p = 0.078	β = − 2.253 (1.592); z (36) = − 1.685; p = 0.092	β = − 0.041; CI [− 0.135 to 0.005]	β = − 0.040 (0.075); z (36) = − 0.553; p = 0.580
Left AHS/Ch. 21/RT in ST	β = 0.000 (0.000); z (38) = 1.758; p = 0.079	β = − 1.781 (1.357); z (38) = − 1.473; p = 0.141	β = − 0.001; CI [− 0.003 to 0.000]	β = − 0.004 (0.003); z (38) = − 1.483; p = 0.138
Left NHS/Ch. 21/RT in ST	β = 0.007 (0.006); z (38) = 0.987; p = 0.324	β = − 1.878 (1.461); z (38) = − 1.389; p = 0.165	β = − 0.013; CI [− 0.065 to 0.012]	β = − 0.082 (0.069); z (38) = − 1.285; p = 0.199
deoxyHb				
Left AHS/Ch. 2/RT in ST	β = 0.000 (0.000); z (39) = − 1.581; p = 0.114	β = 6.522 (5.036); z (39) = 1.562; p = 0.118	β = − 0.001; CI [− 0.006 to 0.000]	*β* = − *0.005 (0.002); z (39)* = − *2.369; p* = *0.018*
Left NHS/Ch. 2/RT in ST	β = − 0.003 (0.003); z (39) = − 1.189; p = 0.235	β = 6.710 (6.091); z (39) = 1.362; p = 0.173	β = − 0.019; CI [− 0.134 to 0.010]	*β* = − *0.098 (0.049); z (39)* = − *2.083; p* = *0.037*
Left AHS/Ch. 5/RT in ST	β = 0.000 (0.000); z (36) = − 1.944; p = 0.052	*β* = *6.349 (3.104); * *z (36) = 2.037; p* = *0.042*	β = − 0.002; CI [− 0.006 to 0.000]	β = − 0.002 (0.003); z (36) = − 0.662; p = 0.508
Right AHS/Ch. 5/RT in ST	β = 0.000 (0.000); z (36) = − 1.480; p = 0.139	*β* = *6.989 (2.621); * *z (36) = 2.689; p* = *0.007*	β = − 0.002; CI [− 0.006 to 0.000]	β = − 0.001 (0.003); z (36) = − 0.198; p = 0.843
Left NHS/Ch. 5/RT in ST	β = − 0.007 (0.005); z (36) = − 1.626; p = 0.104	*β* = *6.473 (3.060); * *z (36) = 2.183; p* = *0.029*	β = − 0.042; CI [− 0.169 to 0.011]	β = − 0.046 (0.031); z (36) = − 0.415; p = 0.678
Right NHS/Ch. 5/RT in ST	β = − 0.005 (0.005); z (36) = − 1.417; p = 0.156	*β* = *6.965 (2.587); * *z (36) = 2.788; p* = *0.005*	β = − 0.034; CI [− 0.147 to 0.009]	β = − 0.028 (0.064); z (36) = − 0.083; p = 0.934
Left AHS/Ch. 5/CA in ST	β = 0.000 (0.000); z (36) = − 1.922; p = 0.055	β = − 133.058 (117.670); z (36) = − 1.223; p = 0.222	β = 0.039; CI [− 0.022 to 0.198]	β = 0.001 (0.089); z (36) = − 0.202; p = 0.840
Right AHS/Ch. 5/CA in ST	β = 0.000 (0.000); z (36) = − 1.483; p = 0.138	β = − 136.131 (119.154); z (36) = − 1.262; p = 0.207	β = 0.030; CI [− 0.019 to 0.170]	β = − 0.006 (0.091); z (36) = − 0.410; p = 0.682
Left NHS/Ch. 5/CA in ST	β = − 0.007 (0.005); z (36) = − 1.609; p = 0.108	β = − 113.039 (115.465); z (36) = − 1.047; p = 0.295	β = 0.737; CI [− 0.752 to 4.501]	β = 0.912 (1.683); z (36) = 0.520; p = 0.603
Right NHS/Ch. 5/CA in ST	β = − 0.005 (0.005); z (36) = − 1.400; p = 0.161	β = − 117.697 (117.911); z (36) = − 1.064; p = 0.287	β = 0.568; CI [− 0.669 to 3.706]	β = 0.691 (1.649); z (36) = − 0.345; p = 0.730
Left AHS/Ch. 7/RT in ST	*β* = *0.000 (0.000); * *z (38) = − 2.180; p* = *0.029*	β = 4.306 (11.759); z (38) = 0.416; p = 0.677	β = − 0.001; CI [− 0.009 to 0.006]	β = − 0.005 (0.004); z (38) = − 1.147; p = 0.251
Left NHS/Ch. 7/RT in ST	β = − 0.005 (0.004); z (38) = − 1.841; p = 0.066	β = 5.213 (7.642); z (38) = 0.739; p = 0.460	β = − 0.026; CI [− 0.174 to 0.070]	β = − 0.091 (0.065); z (38) = − 1.507; p = 0.132
Left AHS/Ch. 14/CA in ST	β = 0.000 (0.000); z (38) = − 1.535; p = 0.125	*β* = − *160.626 (75.764); * *z (38) = − 2.060; p* = *0.039*	β = 0.065; CI [− 0.008 to 0.262]	β = 0.015 (0.072); z (38) = 0.237; p = 0.813
Left NHS/Ch. 14/CA in ST	β = − 0.007 (0.007); z (38) = − 1.327; p = 0.185	*β* = − *154.613 (75.744); * *z (38) = − 1.994; p* = *0.046*	β = 1.151; CI [− 0.384 to 5.134]	β = 0.945 (1.521); z (38) = 0.851; p = 0.395

Significant paths are presented in italic

*AHS* absolute handgrip strength, *CA in ST* percentage of correct answers in Sternberg task, *Ch.* channel, *CI* 95% confidence intervals, *deoxyHb* deoxygenated haemoglobin, *NHS* normalized handgrip strength (normalized to body-mass-index), *RT* reaction time in Sternberg task, *ST* Sternberg task

Regarding Path A, we found that left AHS significantly predict [β = 0.001 (0.000); z (39) = 2.189; p = 0.029] the amplitude of oxyHb in Channel 2 (right Broca; BA 45) and that left AHS significantly predict [β = 0.000 (0.000); z (38) = − 2.180; p = 0.029] the amplitude of deoxyHb in Channel 7 (right FPA; BA 10), although the beta coefficient are very small (i.e., tending towards zero). As shown in Table 4, all other regressions of Path A did not reach statistical significance (p ≥ 0.05).

Regarding the B path, we observed that level of oxyHb in Channel 2 (right Broca; BA 45), Channel 5 (right DLPFC; BA 9), and Channel 10 (middle FPA; BA 10) significantly predict reaction time in Sternberg task (for detailed overview see Table 4). Furthermore, the level of deoxyHb in Channel 5 (right DLPFC; BA 9) predict the reaction time in Sternberg task, whereas level of deoxyHb in Channel 14 (left DLPFC; BA 46) predict the percentage of correct answers in Sternberg task. The other regressions of Path B, which are shown in Table 4, did not reach statistical significance (p ≥ 0.05).

Regarding path AB and C, we found a significant direct effect of left AHS on reaction time in Sternberg task [β = − 0.004 (0.002); z (39) = − 2.175; p = 0.030; oxyHb—Channel 2], although the beta coefficient is very small. However, as the 95% CI for the indirect path included zero, a full mediation effect of the level of oxyHb of Channel 2 (right Broca; BA 45) could not be assumed. Furthermore, we observed a statistically significant direct effect of left AHS [β = − 0.005 (0.002); z (39) = − 2.369; p = 0.018; deoxyHb—Channel 2] and left NHS [β = − 0.098 (0.049); z (39) = − 2.083; p = 0.037; deoxyHb—Channel 2] on reaction time in Sternberg task, but with very small beta coefficients. A full mediation effect of deoxyHb of Channel 2 (right Broca; BA 45) could not be assumed because the 95% CI crossed zero. No other direct or indirect effects were observed to be statistically significant (for detailed overview see Table 4). Taken together, neither oxyHb nor deoxyHb can be considered significant mediators of the relationship between measures of handgrip strength and cognitive performance.

## Discussion

The current study investigated the relationship between measures of handgrip strength, cognitive performance, and cortical hemodynamics. Thereto, we assessed handgrip strength via a handheld dynamometer and recorded cortical hemodynamics during a standardized cognitive test (Sternberg task) using fNIRS.

We observed that a higher cortical activation (objectified by higher level of oxyHb and lower levels of deoxyHb) of distinct parts of the PFC (e.g., FPA and DLPFC; see Tables 2 and 3) is associated with better cognitive performance. This finding is in accordance with the literature reporting that, at least in older adults, a higher level of oxyHb in the PFC during the cognitive testing is associated with better cognitive performance [106]. In this regard, it was also observed that after an acute bout of physical exercise [107–110] and during physical exercise [111], higher levels of oxyHb in distinct parts the PFC (e.g., FPA and DLPFC) are linked to better cognitive performance. In line with these previous observations, our findings buttress the assumption of a positive neurobiobehavioural relationship between cortical hemodynamics and cognitive performance.

Furthermore, we noticed that a higher level of handgrip strength is linked to a higher level of cortical activation (objectified by higher level of oxyHb and lower levels of deoxyHb) in distinct parts of the PFC (see Tables 2 and 3). In the literature, it was observed that a higher level of cardiorespiratory fitness is positively correlated with the magnitude of the oxyHb in the PFC in older adults [112] and negatively correlated with magnitude of deoxyHb in right and left PFC in younger adults [113]. Consequently, our finding supports the general notion that a certain level of muscular strength (e.g., achieved by a regular resistance training) is beneficial for brain health (e.g., cortical hemodynamics) in younger adults [1, 2]. Whether a training-induced increase in (handgrip) strength of younger adults can improve brain health remains speculative because the majority of the available studies in this field of research has focused on older adults [1–3]. By saying that, there is also some evidence available suggesting that training interventions have a limited ability to change measures of handgrip strength in adults [114, 115] although this finding is not universal [116]. Moreover, it is hypothesized that baseline values of (handgrip) strength (e.g., obtained prior to a resistance training) might be a more appropriate indicator regarding health-related outcomes as compared to training-induced changes [117, 118]. Hence, the practical implications of our findings are currently not fully clear which, in turn, calls for further research broaden our knowledge in this direction.

We did not find statistical indices providing compelling evidence that in our cohort of younger adults measures of cortical hemodynamics mediate the relationship between handgrip strength and cognitive performance (see Table 4). The absence of a mediation effect could be related to the absence of a significant correlation between measures of handgrip strength and cognitive performance. The lack of such a correlation contradicts the findings of a previous study [26]. Presumably, these contrary findings are related to the different cognitive tests which have been used. In the study of Choudhary et al. [26], a simple reaction time task was employed whereas we have probed short-term working memory performance with the Sternberg task [44, 45] which is well-established in the field of exercise-cognition science [119–124]. Hence, our findings suggest that handgrip strength is associated with measures of cortical hemodynamics, but that this relationship might not strictly culminate in a superior cognitive performance, at least in our cohort of younger adults. In turn, the absence of a relationship between level of handgrip strength and cognitive functioning suggests that in younger adults, there might be other factors than the level of handgrip strength being important for superior cognitive performance.

Given that previous studies have reported that cortical hemodynamics (e.g., level of oxyHb) are a significant mediator of the relationship between cardiorespiratory fitness and cognition in older adults [30–32], it seems to be promising to investigate if measures of cortical hemodynamics mediate the relationship between handgrip strength and cognitive performance in a cohort of older individuals. This idea is supported by the findings suggesting an association between higher levels of handgrip strength and lesser cognitive decline in older age [12–18]. In this regard, it is also recommended that future studies should aim to assess further fitness dimensions (e.g., muscular fitness, cardiorespiratory fitness, and motor fitness) because it was observed that changes in different fitness dimensions influence the brain differently [125–127].

## Limitations

Despite our presented findings are interesting, this study has limitations which warrant further discussion. Firstly, even if we have account for the confounding influence of superficial blood flow by a short-separation channel regression, it is recommended that future studies should consider to quantify additional physiological parameters (e.g., blood pressure, respiration rate, skin conductance) to assess the influence of systemic physiological changes more comprehensively (also referred to as ‘systemic physiological augmented fNIRS’ [128–133]) which, in turn, can reduce the risk of ‘false positive’ findings in fNIRS studies [80]. Secondly, although the sample size in the current study is in the range of comparable investigations [30], it is relatively small. In this context, it is also important to acknowledge that our findings are not generalizable because we only included young right-handed individuals in order to avoid laterality effects. Thirdly, we did not perform multiple comparison adjustments. However, there is an ongoing discussion about when and how it would be necessary to adjust for multiple comparisons [134–136] and it is stated that in exploratory studies, multiple comparison adjustments are not strictly required [135]. Fourthly, as the findings of our cross-sectional study are based on correlational analyses, it is not possible to derive strong (causal) conclusions. Cognizant of these limitations, further cross-sectional and interventional studies with a larger sample size are necessary to confirm (or rebut) our findings and to investigate whether those are generalizable to other cohorts (e.g., older adults without and with cognitive impairments).

## Conclusion

In summary, our findings show promising evidence for a positive neurobehavioral relationship between cortical hemodynamics and cognitive performance. Moreover, we complement the existing literature by adding that in younger adults higher levels of handgrip strength are linked to more pronounced cortical hemodynamics which imply that muscular strength influences brain health positively. However, our work also showed that in younger adults such a positive effect on a parameter of brain health does not necessarily benefit cognitive performance as we did not find convincing evidence for a relationship between handgrip strength and cognitive performance or that cortical hemodynamics mediate this relationship. Considering that such relationships might change as function of age and disease, further research should aim to investigate whether our findings are also generalizable to different cohorts (e.g., older adults) and whether different fitness dimensions influence cognitive performance and cortical hemodynamics differently.

## Supplementary Information

**Additional file 1: Table S1.** Detailed overview of the probe placement which shows the exact position of the sources and detectors in the 10–20 EEG system, the MNI coordinates (X, Y, Z), the corresponding anatomical landmarks (e.g., dorsolateral prefrontal cortex) and the distance between sources and detectors. Please note that this output was generated by using the fOLD-software [68]

## Data Availability

The public sharing of the data from this trial has been restricted by the local Research Ethics Committee in order to protect participants’ privacy. Data are therefore only available on reasonable request from the corresponding author.

## Abbreviations

AHS: Absolute handgrip strength
A.U.: Arbitrary unit
BA: Broadman area
bpm: Beats per minute
BDI-II: Becks Depression Inventory II
BMI: Body mass index
BSA-F: “Bewegungs- und Sportaktivitätsfragebogen”
CA: Correct answers
Ch.: Channel
CI: Confidence interval
cm: Centimeter
CRF: Cardiorespiratory fitness level
CV: Coefficient of variation
deoxyHb: Deoxygenated hemoglobin
DLPFC: Dorsolateral prefrontal cortex
EEG: Electroencephalography
EHI: Edinburgh Handedness Inventory
fNIRS: Functional near-infrared spectroscopy
fOLD: FNIRS Optodes’ Location Decider
FPA: Frontopolar area
GLM: General linear model
HF: High frequency
HRF: Hemodynamic response function
IQR: Interquartile range
kg: Kilogram
LF: Low frequency
LSC: Long-separation channel
lt.: Left
MBLL: Modified Beer–Lambert law
md.: Middle
mm: Millimeter
NHS: Normalized handgrip strength
oxyHb: Oxygenated hemoglobin
PA: Physical activity
PE: Physical exercise
PFC: Prefrontal cortex
PSQI: Pittsburgh Sleep Quality Index
r_p_: Partial correlation coefficient
RT: Reaction time
s: Seconds
SSC: Short-separation channels
ST: Sternberg task
TMT: Trail Making Test
VLF: Very low frequency
${\text{VO}}_{{2_{\max .} }}$: Maximal oxygen uptake
