# Supplementary material for: Cortical hemodynamics as a function of handgrip strength and cognitive performance: a cross-sectional fNIRS study in younger adults
Source: BMC Neurosci. 2021 Feb 15;22:10. doi: 10.1186/s12868-021-00615-6 (PMC7885414; doi:10.1186/s12868-021-00615-6)
Supplement: Supplementary file 1 — Additional file 1: Table S1. Detailed overview of the probe placement which shows the exact position of the sources and detectors in the 10–20 EEG system, the MNI coordinates (X, Y, Z), the corresponding anatomical landmarks (e.g., dorsolateral prefrontal cortex) and the distance between sources and detectors. Please note that this output was generated by using the fOLD-software [68] [file 12868_2021_615_MOESM1_ESM.docx]

**Table S1.** Detailed overview of the probe placement which shows the exact position of source and detectors in the 10-20 EEG system, the MNI coordinates (X, Y, Z), the corresponding anatomical landmarks (e.g., dorsolateral prefrontal cortex) and respective specificity, as well as the distance between source and detector. Please note that this output was generated by using the fOLD-software (G. A. Zimeo Morais, J. B. Balardin, and J. R. Sato, “fNIRS Optodes' Location Decider (fOLD): A toolbox for probe arrangement guided by brain regions-of-interest,” Scientific reports, vol. 8, no. 1, p. 3341, 2018.).

| **Channel** | **Position** | **Source** | **Detector** | **X (mm)** | **Y (mm)** | **Z (mm)** | **Landmark** | **Specificity (%)** | **Distance (mm)** |
| --- | --- | --- | --- | --- | --- | --- | --- | --- | --- |
| 1 | F4-F2 | F4 | F2 | 30 | 40 | 41 | 9 - Dorsolateral prefrontal cortex | 68,36664315 | 30 |
|  |  |  |  |  |  |  | 46 - Dorsolateral prefrontal cortex | 22,39597886 |  |
|  |  |  |  |  |  |  | 8 - Includes Frontal eye fields | 5,568764573 |  |
|  |  |  |  |  |  |  |  |  |  |
| 2 | F4-F6 | F4 | F6 | 46 | 38 | 24 | 45 - pars triangularis Broca's area | 70,67253504 | 30 |
|  |  |  |  |  |  |  | 46 - Dorsolateral prefrontal cortex | 23,4516285 |  |
|  |  |  |  |  |  |  |  |  |  |
| 3 | AF8-F6 | AF8 | F6 | 48 | 46 | 5 | 45 - pars triangularis Broca's area | 43,87699256 | 33 |
|  |  |  |  |  |  |  | 46 - Dorsolateral prefrontal cortex | 43,17605645 |  |
|  |  |  |  |  |  |  | 47 - Inferior prefrontal gyrus | 5,07367444 |  |
|  |  |  |  |  |  |  |  |  |  |
| 4 | AF8-Fp2 | AF8 | Fp2 | 34 | 59 | -2 | 10 - Frontopolar area | 31,0841122 | 30 |
|  |  |  |  |  |  |  | 11 - Orbitofrontal area | 30,472822 |  |
|  |  |  |  |  |  |  | 46 - Dorsolateral prefrontal cortex | 20,4016318 |  |
|  |  |  |  |  |  |  |  |  |  |
| 5 | AF4-F2 | AF4 | F2 | 22 | 52 | 33 | 9 - Dorsolateral prefrontal cortex | 51,5248078 | 44 |
|  |  |  |  |  |  |  | 46 - Dorsolateral prefrontal cortex | 26,41541438 |  |
|  |  |  |  |  |  |  | 10 - Frontopolar area | 18,40538603 |  |
|  |  |  |  |  |  |  |  |  |  |
| 6 | AF4-F6 | AF4 | F6 | 40 | 50 | 16 | 46 - Dorsolateral prefrontal cortex | 47,36091434 | 45 |
|  |  |  |  |  |  |  | 45 - pars triangularis Broca's area | 30,60393436 |  |
|  |  |  |  |  |  |  | 10 - Frontopolar area | 18,97467131 |  |
|  |  |  |  |  |  |  |  |  |  |
| 7 | AF4-Fp2 | AF4 | Fp2 | 25 | 63 | 9 | 10 - Frontopolar area | 68,78124608 | 28 |
|  |  |  |  |  |  |  | 11 - Orbitofrontal area | 21,56708753 |  |
|  |  |  |  |  |  |  | 46 - Dorsolateral prefrontal cortex | 6,609548215 |  |
|  |  |  |  |  |  |  |  |  |  |
| 8 | AF4-AFz | AF4 | AFz | 13 | 61 | 24 | 10 - Frontopolar area | 72,46582734 | 36 |
|  |  |  |  |  |  |  | 9 - Dorsolateral prefrontal cortex | 16,97337784 |  |
|  |  |  |  |  |  |  | 46 - Dorsolateral prefrontal cortex | 7,97503285 |  |
|  |  |  |  |  |  |  |  |  |  |
| 9 | Fpz-Fp2 | Fpz | Fp2 | 13 | 67 | 0 | 10 - Frontopolar area | 54,45817975 | 31 |
|  |  |  |  |  |  |  | 11 - Orbitofrontal area | 44,84702617 |  |
|  |  |  |  |  |  |  |  |  |  |
| 10 | Fpz-Afz | Fpz | AFz | 1 | 64 | 14 | 10 - Frontopolar area | 87,47712753 | 40 |
|  |  |  |  |  |  |  | 9 - Dorsolateral prefrontal cortex | 5,080149043 |  |
|  |  |  |  |  |  |  |  |  |  |
| 11 | Fpz-Fp1 | Fpz | Fp1 | -12 | 67 | 0 | 10 - Frontopolar area | 54,49683736 | 30 |
|  |  |  |  |  |  |  | 11 - Orbitofrontal area | 44,89777168 |  |
|  |  |  |  |  |  |  |  |  |  |
| 12 | AF3-AFz | AF3 | AFz | -12 | 62 | 23 | 10 - Frontopolar area | 75,76225222 | 36 |
|  |  |  |  |  |  |  | 9 - Dorsolateral prefrontal cortex | 14,5484687 |  |
|  |  |  |  |  |  |  | 46 - Dorsolateral prefrontal cortex | 8,053792322 |  |
|  |  |  |  |  |  |  |  |  |  |
| 13 | AF3-Fp1 | AF3 | Fp1 | -24 | 63 | 9 | 10 - Frontopolar area | 69,63399373 | 27 |
|  |  |  |  |  |  |  | 11 - Orbitofrontal area | 20,11557888 |  |
|  |  |  |  |  |  |  | 46 - Dorsolateral prefrontal cortex | 8,793079404 |  |
|  |  |  |  |  |  |  |  |  |  |
| 14 | AF3-F5 | AF3 | F5 | -39 | 50 | 17 | 46 - Dorsolateral prefrontal cortex | 49,33992409 | 44 |
|  |  |  |  |  |  |  | 45 - pars triangularis Broca's area | 32,11689876 |  |
|  |  |  |  |  |  |  | 10 - Frontopolar area | 16,03613047 |  |
|  |  |  |  |  |  |  |  |  |  |
| 15 | AF3-F1 | AF3 | F1 | -23 | 52 | 32 | 9 - Dorsolateral prefrontal cortex | 48,43964622 | 44 |
|  |  |  |  |  |  |  | 46 - Dorsolateral prefrontal cortex | 32,04248075 |  |
|  |  |  |  |  |  |  | 10 - Frontopolar area | 16,88767141 |  |
|  |  |  |  |  |  |  |  |  |  |
| 16 | AF7-Fp1 | AF7 | Fp1 | -33 | 59 | -2 | 11 - Orbitofrontal area | 32,71174196 | 30 |
|  |  |  |  |  |  |  | 46 - Dorsolateral prefrontal cortex | 25,27140585 |  |
|  |  |  |  |  |  |  | 10 - Frontopolar area | 25,12699295 |  |
|  |  |  |  |  |  |  | 47 - Inferior prefrontal gyrus | 13,10460546 |  |
|  |  |  |  |  |  |  |  |  |  |
| 17 | AF7-F5 | AF7 | F5 | -47 | 46 | 6 | 45 - pars triangularis Broca's area | 48,7862379 | 33 |
|  |  |  |  |  |  |  | 46 - Dorsolateral prefrontal cortex | 43,20372761 |  |
|  |  |  |  |  |  |  |  |  |  |
| 18 | F3-F5 | F3 | F5 | -46 | 39 | 26 | 45 - pars triangularis Broca's area | 72,56408005 | 29 |
|  |  |  |  |  |  |  | 46 - Dorsolateral prefrontal cortex | 21,83837451 |  |
|  |  |  |  |  |  |  |  |  |  |
| 19 | F3-F1 | F3 | F1 | -31 | 39 | 41 | 9 - Dorsolateral prefrontal cortex | 66,60628263 | 29 |
|  |  |  |  |  |  |  | 46 - Dorsolateral prefrontal cortex | 24,84615413 |  |
|  |  |  |  |  |  |  |  |  |  |
| 20 | Fz-F2 | Fz | F2 | 10 | 41 | 50 | 9 - Dorsolateral prefrontal cortex | 68,93306313 | 29 |
|  |  |  |  |  |  |  | 8 - Includes Frontal eye fields | 28,89129929 |  |
|  |  |  |  |  |  |  |  |  |  |
| 21 | Fz-AFz | Fz | AFz | 2 | 50 | 39 | 9 - Dorsolateral prefrontal cortex | 61,76828691 | 40 |
|  |  |  |  |  |  |  | 10 - Frontopolar area | 20,26301996 |  |
|  |  |  |  |  |  |  | 8 - Includes Frontal eye fields | 12,14994939 |  |
|  |  |  |  |  |  |  | 32 - Dorsal anterior cingulate cortex | 5,083608014 |  |
|  |  |  |  |  |  |  |  |  |  |
| 22 | Fz-F1 | Fz | F1 | -9 | 41 | 50 | 9 - Dorsolateral prefrontal cortex | 63,16113601 | 29 |
|  |  |  |  |  |  |  | 8 - Includes Frontal eye fields | 34,73195285 |  |
